# *Pathogens*: A New Open Access Journal Serving All Those Interested in Infectious Disease

**DOI:** 10.3390/pathogens1010001

**Published:** 2011-09-29

**Authors:** Lawrence S. Young

**Affiliations:** College of Medical and Dental Sciences, University of Birmingham, Birmingham, B15 2TT, UK; E-Mail: l.s.young@bham.ac.uk; Website: http://medweb4.bham.ac.uk/ssp/Printable.aspx?id=1900; Tel.: +44-1214-146876

Infection ranks alongside cardiovascular disease as the major cause of human death across the world. Word Health Organization data for 2002 shows that 26% of all deaths, almost 15 million in number, were due to infectious disease with HIV/AIDS, TB and malaria being the top three responsible infections. A significant proportion of these deaths were due to lower respiratory infections and diarrheal diseases in children. The worldwide morbidity associated with infectious disease is incalculable. When considered along with the consequences of infection in animals, it is hard to imagine any other disease that has such a significant impact on our lives―on health systems, on agriculture and on world economics.

Our understanding of the agents responsible for infections―bacteria, fungi, parasites, prions and viruses―has an interesting history that heralds the great developments in modern biology and demonstrates how an understanding of disease pathogenesis can lead to successful prophylactic and therapeutic interventions. Van Leeuwenhoek’s first observation of bacteria under the light microscope, John Snow’s investigations tracing the source of a cholera epidemic in Victorian London’s Soho and Pasteur’s vaccines for rabies and anthrax contributed to an acceptance of the germ theory of disease and to the rational, scientific application of this knowledge to develop innovative disease control measures ranging from hygienic practices to antibiotics.

The study of pathogens has provided fundamental insights into the basic molecular mechanisms underlying cell biology. Whether it’s the processes responsible for generating innate and adaptive immune responses, the cell signaling pathways governing cell cycle control or the deregulated transcriptional programs driving oncogenesis, pathogens have provided invaluable tools for understanding the underlying cell and molecular biology. This continues to be the case and there is no area of modern biology that is not influenced by our deepening appreciation of the behavior and impact of pathogens.

*Pathogens* aims to capture the significance and relevance of infectious disease in an open access journal that will provide a forum for publications covering the full range from regular research papers and reviews, to short notes and discussion articles. We will encourage the comprehensive publication of experimental detail to promote the full discussion of new information and to ensure that new approaches and techniques are shared with the research community. We also hope to publish themed issues and would welcome suggestions for research areas that would benefit from a more focused consideration. We hope that *Pathogens* will become a vibrant forum for discussion, book reviews, the announcement of conferences and relevant advertisements. We are privileged to be served by an outstanding Editorial Board who represent the breadth of the subject area and will provide a wealth of experience in supporting our continued understanding of infectious disease. I hope that you will join us in this new initiative and consider *Pathogens* as an important vehicle in which to publish your research.

